# Theory of Self- vs. Externally-Regulated Learning^TM^: Fundamentals, Evidence, and Applicability

**DOI:** 10.3389/fpsyg.2017.01675

**Published:** 2017-09-29

**Authors:** Jesús de la Fuente-Arias

**Affiliations:** ^1^Department of Psychology, Faculty of Psychology, Developmental and Educational Psychology, University of Almería, Almería, Spain; ^2^Department of Psychology, Facultad de Ciencias Sociales y Humanidades, Universidad Autónoma de Chile, Santiago de Chile, Chile

**Keywords:** personal self-regulation (SR), externally-regulated learning (ERL), self-regulated learning (SRL), regulatory teaching (RT), theory review

## Abstract

The *Theory of Self- vs. Externally-Regulated Learning*^TM^ has integrated the variables of SRL theory, the DEDEPRO model, and the 3P model. This new Theory has proposed: (a) in general, the importance of the cyclical model of individual self-regulation (SR) and of external regulation stemming from the context (ER), as two different and complementary variables, both in combination and in interaction; (b) specifically, in the teaching-learning context, the relevance of different types of combinations between levels of self-regulation (SR) and of external regulation (ER) in the prediction of self-regulated learning (SRL), and of cognitive-emotional achievement. This review analyzes the assumptions, conceptual elements, empirical evidence, benefits and limitations of *SRL vs. ERL Theory*. Finally, professional fields of application and future lines of research are suggested.

## Introduction

The results of research on the topics of *Self-Regulation* and *Self-Regulated Learning* have been abundant in recent years. However, these lines of investigation have followed parallel paths and contexts. So while the *Self-Regulation* (SR) construct has belonged mainly to contexts of clinical psychology and of health care (Howell and Buro, 2011; Clark, 2013; Clark et al., 2014), the psychological construct of *Self-Regulated Learning* (SRL) has appeared more typically in psychoeducational settings (Zimmerman and Schunk, 1989, 2011; Hadwin et al., 2001, 2010; Winne, 2001, 2005; Torrano and González-Torres, 2004; Winne and Hadwin, 2008; Bembenutty et al., 2014; Whitte and Dibenedetto, 2015; Paulino and Da Silva, 2016; Panadero, 2017). Some authors have already asserted the need for a meta-theoretical convergence of the two lines of research (Boekaerts et al., 2005; Karoly et al., 2005) as they are thought to be different manifestations of the same psychological variable. However, this integrating proposal is still to be brought to fruition. Our theoretical proposal attempts to contribute to it.

For this reason, the *objective* of this review is to present *SRL vs. ERL Theory*^TM^ (de la Fuente, 2015) as a new theoretical formulation, and as a way of bringing about this improved conceptual step, at different levels: (1) Fundamentals, or theoretical principles, and their application to the psycho-educational context; (2) Evidence and limitations; (3) General and specific applicability; and (4) Conclusions and future research.

## Fundamentals

### Theoretical Principles

The conceptual foundation of this Theory is built on the following theoretical assumptions.

#### Principle 1. The Self-regulatory, A-regulatory, or De-regulatory Behavior As Personal Characteristic:

This theoretical model furthers the definitions of each type of behavioral regulation of the individual, on a behavioral continuum.

(1) *Self-Regulation* (SR) may be considered as the degree of the person’s *positive proactivity*, that is to say, in his active and adequate management of the regulation of his conduct (Brown, 1998). Previous research has shown that people can have different degrees of personal self-regulation (high-medium-low), alluding to the degree and to the quantity of behaviors they employ in order to exercise their own behavioral regulation (de la Fuente et al., 2015c; Zapata, 2013, Unpublished).
(2) *A-Regulation* (AR) may be defined conceptually as the lack of proactivity or the absence of self-regulatory behaviors in the person. Conceptually, they are equivalent to the concept of *reactivity* (Zimmerman and Labuhn, 2012). In this case the individual is at the mercy of the externally-regulatory system of the context.
(3) *De-regulation* (DR) should be understood as the degree of *negative proactivity*, that is, of active and inadequate management to regulate one’s behavior. As may be seen, this de-regulation may have advantageous spin-offs because individuals avoid the effort involved in proactive self-regulation, for example, strategies of self-impediment (Valle et al., 2007) or of procrastination (Clariana, 2013; Balkis and Duru, 2017). These behavior types are shown in **Table 1**.

**Table 1:** Conceptual continuum and typologies of each Self-Regulatory Behavior.

Characteristics of the person	Self-regulation (SR) High- moderate- low POSITIVE PRO-ACTIVITY (+1)	A-regulation (AR) No regulation RE-ACTIVITY (0)	De-regulation (DR) Low- moderate- high NEGATIVE PRO-ACTIVITY (-1)
	*Before* Self-analysis of tasks Self-defines goals Self-motivation	*Before* No analysis of tasks No goals No motivation	*Before* Erroneous self-analysis Erroneous goals Self-demotivation
	*During* Self-observation Self-analysis Self-correction	*During* No self-observation No supervision No self-correction	*During* Self-distraction Cognitive self-avoidance Self-impediment Procrastination
	*After* Self-reflection Self-attributions Positive self-affects	*After* No reflection No attributions No affects	*After* Erroneous self-assessment Erroneous self-attributions Negative self-affect

**Type of activity**	**Self-regulatory (SR)** **High-moderate-low** **PRO-ACTIVITY (+)**	**A-Regulatory (AR)** **No regulation** **RE-ACTIVITY (0)**	**De-Regulatory (DR) Low-moderate- high PRO-ACTIVITY (–)**

Academic	Self-regulated learning	No norms/limits	Self-induction impediment
Road safety	Self-regulation in driving	No norms/limits	Self-induction of risks
Health	SR in health	No norms/limits	Self-induction of excesses
TV	SR in TV	No norms/limits	Self-induction of excesses
Family	SR in family	No norms/limits	Self-induction of risks
Technology of Information and Communication (TIC)	SR in TIC	No norms/limits	Self-induction of excesses
Sexual	SR in risky sexual behavior	No regulation	Self-induction of risks
Violence	SR in harmonious relations	No norms/limits	Self-induction of excesses
Spouse/partner	SR in interaction	No regulation	Self-induction of excesses

#### Principle 2. The Externally-Regulatory, A-regulatory, or De-regulatory Nature of the Context:

This theoretical model considers the *context* as the set of situational stimuli that can make probable the directionality of a behavior, in interaction with the subject, being these of real, virtual or symbolic type. They require some degree of structuring between the set of stimuli, which can include people, material elements, signals or virtual. The context provides individual, temporal, activity, social, and location information (Zimmermann et al., 2007). Context can also be:

(1) *External Self-Regulatory* (ESR). The context promotes positive or adequate proactivity, or clearly fosters self-regulation. In this context there are numerous external signs or encouragements which promote and make self-regulated behavior more likely at the beginning, during, and at the end of all behavioral acts. These occur through *antecendents* (patterns, norms, limits, expectations of success in self-regulation, value of self-regulation) and through contextual *consequences* (positive and negative contingencies favoring self-regulation, adaptation...). Highly predictable of positive events are a feature of this context.
(2) *External A-Regulatory* (EAR). The context does not promote external self-regulation, or de-regulation. In this context there are no external signs or encouragements to make self-regulated behavior or de-regulated behavior more likely at the beginning, during, or at the end of the action. An *a-regulatory* context entails that the individual must engage in a moderate level of self-regulatory behavior, as there are no contextual elements to direct it one way or the other. Highly unpredictable events are a feature of this context.
(3) *External De-regulatory* (EDR), actively promoting de-regulation. The context promotes non-positive, inadequate, or negative proactivity. In this context, there are many external signs which make *de-regulated* behavior more likely, favoring active de-regulation at the beginning, during, and at the end of the behavioral act. This kind of behavior also occurs through contextual antecedents (modeling, rules, limits, expectations of success in self-regulation, value of self-regulation...) and also through contextual consequents (positive and negative contingencies, molding…), which favor de-regulation. This kind of context means that the individual needs to make a great effort to engage in self-regulation. Highly predictable of negative events are a feature of this context. See **Table 2**.

**Table 2:** Conceptual continuum of the Externally-Regulatory Learning (ERL) context dimension.

Characteristics of the context	External self-regulation (ESR) High-moderate-low POSITIVE PROACTIVITY (+1)	External A-regulation (EAR) No regulation RE-ACTIVITY (0)	External De-regulation (EDR) Low-moderate- high NEGATIVE PRO-ACTIVITY (-1)
	*Before* Presents analysis of tasks Suggests adjusted goals Suggests self-motivation	*Before* Does not present tasks Does not propose goals Does not induce motivation	*Before* Erroneous tasks Erroneous goals (self-impediment) Induces demotivation
	*During* Promotes self-observation Promotes self-analysis Promotes Self-correction	*During* No self-observation No supervision No self-correction	*During* Promotes self-distraction Cognitive self-avoidance Self-impediment Procrastination
	*After* Promotes self-reflection Promotes adjusted self-attributions Promotes positive adjusted self-affects	*After* No reflection No attributions No affects	*After* Promotes erroneous self-assessment Erroneous self-attributions Promotes maladjusted self-affects

**Type of context**	**Externally-regulatory** **High moderate low**	**A-regulatory** **No regulation**	**De-regulatory** **Low moderate high**

Academic	Effective/regulatory teaching (RT)	Laissez-faire	Stressful teaching
Road safety	Correct traffic signs	No traffic signs	Road inducing speeding
Health	Norms/limits of consumption	No norms/consequences	Negative drinking contexts
TV	Norms/limits	No norms/limits	Negative TV contexts
Family	Authoritative/democratic	Permissive/laissez-faire	Liberal/promoting de-regulation
Technology of Information and Communication (TIC)	Regulatory norms/limits	No norms/limits	Negative contexts
Sexual	Regulatory norms/consequences	No norms	Contexts which induce lack of control
Violence	Contexts with norms/values	No norms/values	Contexts which induce violence
Partner	Consensual interactions, norms in agreements	No norms	Changeable, unpredictable norms

#### Principle 3. Cyclical Phases (before-during-after) in the Person and in the Context:

This principle assumes that three cyclical and recurring phases (before, during, and after) occur both in the *individual* and in the *context*, making it likely that characteristics of the context contribute or not to the individual’s self-regulation.

#### Cyclical Phases in the Personal Self-regulation

In the *Personal Self-Regulation* (SR), this assumes the same conception and principles as the theory of Self-Regulated Learning (SRL) at each phase, described above (Zimmerman, 2001). This theoretical model (Zimmerman, 2000, 2008; Schunk, 2001) involves three substantial advances: (a) the importance of learners interpreting personal feedback, in terms of controllable processes, (b) the interactivity between metacognitive and motivational intra-processes, and (c) that all these events and processes may be recorded and modified as they occur in real time. Zimmerman and Labuhn (2012, p. 399) have summarized the characteristics of this psychological construct: “(a) self-regulation is conceived as a key mediator between a mental skill and the acquisition of an academic skill. More specifically, it refers to the *self-directive process* with which learners transform their mental abilities into academic ones; (b) research into self-regulated learning does not limit this concept to an individual characteristic of ways of learning, but rather it involves ways of learning, and systems of help among peers and teachers.” Self-Regulated Learning takes on board self-motivational and metacognitive processes. Studies into SRL suggest that self-regulated students participate more actively in the meta-cognitive, meta-motivational and meta-behavioral senses in the learning process, and that they generate more metacognitive strategies, positive expectations of self-efficacy, modification of inefficient actions, and more adjustment of goals. All definitions of self-regulated learning imply the use of direction toward goals (before), the use of three self-regulation properties (while), and as a result, students are more deeply involved in their academic learning (after). Self-regulated learning is cyclical in nature and is dependent on external feedback, especially during situations of sustained effort and when goals must be maintained over time.

##### Preparation stage

Referring to the *analysis of tasks* and to *self-motivational* ideas (self-efficacy, interest in or value of the task, and direction toward the goal). The *analysis of tasks* presupposes an effort of analysis and a breakdown of the task into component parts. It defines the elements of this phase (setting of goals and planning). Proactive students, who are extremely capable of analysis in this area, will clearly and thoroughly define their goals. By contrast, *reactive* and superficial students will carry out a superficial analysis of the task, and define vague goals. Highly *proactive* students will conduct more effective planning, with strategies of cognitive involvement, and with affective and motor control. On the other hand, *reactive* learners will have less knowledge about their strategies, will use less precise learning methods, and will force themselves to concentrate.

Like the task analysis, the definition of *strategic objectives*, within the planning, implies the engagement of the students, that is to say, a high level of meta-motivation. *Proactive* students will employ diverse motivational resources, such as ideas of self-confidence, interest and value of the task, and learning goals. By contrast, *reactive* students will use strategies of self-impediment, and will have low expectations, desire for immediate results, and fear of bad outcomes.

##### Control of execution phase

Through the use of *self-control* (use of strategies) and *self-observation* (by meta-cognitive supervision strategies and self-registering). *Self-control “*refers to the strategic use of various cognitive, motivational and behavioral strategies to guide learning” (Zimmerman and Labuhn, 2012, p. 403). At this stage, *proactive* students implement suitable strategies that they had planned previously, while reactive ones carry out tasks without explicit strategies or guiding methods. *Self-observation* refers to methods used to supervise execution. Metacognitive supervision skills create a mental image of execution and self-reminders to the process and to the product, for example, by means of a graphic record of daily tasks. The self-reminder is an advantage that *proactive* learners have because it increases the reliability of the execution, as well as time spent on self-observations. *Reactive* learners find self-observation difficult because they rarely make a previous plan of targets or a plan that would help them focus their attention, and in the supervision of results the number of personal processes and abilities involved exceeds their limited capacity for memory in the short-term. They do not carry out metacognitive supervision or one which would require monitoring effort at key moments in the process.

##### Self-reflection phase

By means of *self-judgments* (self-assessment and causal attributions) and *self-reaction* (positive, neutral, negatives). *Self-judgments* refer to self-assessments of the efficiency of executions during learning tasks, associated with their *causal attributions*. *Proactive* students are guided at the planning stage by their specific targets, and carry out self-assessments associated with the result, based on them. *Reactive* students carry out few self-assessments, or if they do, these will be aided by social comparison or by the judgment of important persons. Self-assessment judgments made by these students will probably be associated more with causal attributions related to lack of ability, which suggests an attribution with a non-controllable cause. On the other hand, *proactive* students make their self-evaluations based on goals chosen by themselves, typically attributing their errors to ineffective learning strategies or to strategies related to poor ability, both of which are controllable. In general, students choose subjects which give them satisfaction and arouse positive emotions, and avoid those which do not. *Reactive* students attribute their errors to causes beyond their control, feel dissatisfaction and lack involvement in learning. *Proactive* learners put their errors down to controllable causes, have positive emotional states, and sustain their efforts to learn.

The *self-reactions* are inferences about the ideas students make in relation to the effort they have made or their need to continue doing the task. *Proactive* learners, with favorable attributions and high levels of satisfaction, make more adaptive inferences, recognizing their own errors, and changing or modifying their strategies. *Reactive* students make more unfavorable attributions, have lower levels of satisfaction, make more inferences that are protective of themselves, of their dissatisfaction, and of aversive effects, and are prone to more abandonment, task avoidance, lack of cognitive involvement, and apathy. These self-reactions complete the self-regulatory model. The high levels of positives emotions enjoyed by *positive proactive* students involve them in several forms of meta-motivation to continue making an effort and to maintain their ideas of self-efficacy. What is more, this entails adaptive inferences associated with learning of planning and of goals necessary for the future. Nevertheless, the low levels of positives emotions (*reactive students*) and high level of negatives emotions (*negative proactive students*) experienced reduce their motivation to persist and their possibilities of adaptation, as well as the quality of their efforts to learn.

#### Cyclical Phases in the Context

In the *context, t*his assumes that context can work as an enhancer of the three self-regulation phases (ERL), but from the outside:

##### Promotion of preparation phase

This refers to the external drive coming from the analysis of tasks and from self-motivational ideas (self-efficacy, interest or value of the task and direction toward the goal). Context can promote the analysis of tasks, which implies encouraging and modeling the effort of analysis and of breaking down the task into parts, defining the elements of this phase (goal-setting and planning). Proactive or externally-regulatory contexts, and in the field of education, parents or teachers, who are highly skilled at task analysis, will clearly define the tasks and make target achievement much more likely. By contrast, external a-regulatory contexts, which foster reactivity and superficiality in students, will promote a superficial task analysis, and define vague objectives. Externally-regulatory or *proactive contexts* will likely give rise to more effective planning, with strategies of cognitive involvement, and of motor and affective control. However, *a-regulatory* or *de-regulatory* contexts will be associated with poorer knowledge of learning strategies, with less precise learning methods and will not promote students’ attention or concentration. Like task analysis, goal setting and strategy planning requires a facilitating context of personal involvement and persistence, as well as a high level of self-motivation. *Externally positive* and proactive contexts (EPR) resort to diverse motivational resources, favoring ideas of self-efficacy, interest and value of the task, and learning objectives. Externally *A-regulatory* (EAR) or reactive, and externally *de-regulatory* context (EDR) or negative proactive, however, are associated with the use of self-impediment strategies, low expectations, desire for immediate results, and fear of poor outcomes.

##### Promotion of self-control in execution phase

Through the use of hetero-regulatory strategies, this phase is associated with self-control (which promotes the use of decision-taking strategies) and with self-observation (which employs meta-cognitive strategies of supervision and self-recall, such as writing reminders to improve self-observations and self-instructions). The contextual promotion refers to the procedural use of diverse cognitive, motivational and behavioral strategies in order to *promotion of self-control.* At this stage, instructors and regulatory and proactive contexts encourage learners to implement suitable strategies that have been planned previously, while *reactive* contexts promote tasks without explicit strategies or guiding methods.

The contextual promotion of self-observation refers to methods and strategies which foster and oversee execution. Proposals made by the teacher or enhanced by the context for using metacognitive abilities to further supervision, conjures up a mental image of the execution and encourages students to devise self-reminders of the process and of the product, for example, to write a note of the daily tasks to be done. Also, taking time to make *self-reminders* is one advantage of *regulatory/proactive contexts* because it increases the reliability of the execution, as well as time devoted to self-observations. *Reactive* (*a-regulatory)* and *negative proactive context (de-regulatory*) contexts do not aid self-observation because rarely do they involve previous planning of goals or outline a clear schedule of objectives which would help focus learners’ attention, and in supervision of outcomes the number of personal processes and skills involved exceeds the limited capacity for memory in the short term. What is more, these contexts do not promote metacognitive supervision or monitoring of effort on the part of the learner, at key points in the process.

##### Promotion of self-reflection phase

By means of the external promotion of the use of adjusted *self-judgments* (self-assessment and causal attributions) and adjusted *self-reactions*, such as self-satisfaction, adaptive reactions and defensive reactions (Zimmerman and Labuhn, 2012). Through adjusted feedback, dialog, and affective persuasion, the context – or the teacher – may successfully promote these kinds of behaviors. Inductions of *self-judgment* refer to effective self-evaluations in each one of the learning executions, associated with *causal attributions* of the same fostered by the context. *Proactive* (*hetero-regulatory*) contexts guide learners toward their specific goals from the planning stage, as well as promote self-assessments later associated with results, based on the same. *Reactive* contexts will foster few adjusted self-assessments (*a-regulatory*) or, if students do make self-assessments, these will be based on social comparison or on the judgment of significant others, in some cases with negative (*de-regulatory*) messages. Consequently, students’ value judgments in these contexts will most probably be associated with causal attributions, with a lack of ability, which suggests an attribution to a non-controllable cause. By contrast, *proactive* (*hetero-regulatory*) contexts will *promote* learners’ evaluations of themselves taking into account their own aims and objectives, and they will put their mistakes and omissions down to inefficient learning strategies, or to strategies associated with poor capabilities, both of which may be controlled.

The external inductions of self-judgments about learning are associated with two kinds of fostering of *self-reaction*: self-satisfaction and adaptive inferences (Zimmerman and Labuhn, 2012). Bringing about feeling of *self-satisfaction*, which are one’s perceptions of fulfillment or of disappointment, is linked to emotional responses while observing one’s performance, is typical of externally-regulatory contexts. The persons choose contexts (and academic subjects) which arouse in them satisfaction and positive emotions, and avoid ones which make them have negative reactions. *Reactive* (*de-regulatory*) contexts *promote* attributions of errors to controllable causes and emotional states of dissatisfaction to the lack of involvement to learn. *Proactive* (*hetero-regulatory*) contexts *induce* attibutions of errors to controllable causes, as well as positive emotional states in students, who will sustain their efforts to learn. Neutral contexts (*a-regulatory*) neither promote nor induce any self-reaction tendency.

The external promotion of diverse kinds of self-reaction, refer to induction the ideas that students have about their efforts or about their need to persevere at the task. *Proactive (positive externally-regulatory*) contexts give rise to favorable attributions and to high levels of satisfaction, as well as to more adaptive inferences in students, who recognize their mistakes and change or modify their strategies. *Reactive (a-regulatory)* and *de-regulatory (negative externally-regulatory*) contexts bring about more unfavorable attributions, lower satisfaction levels, more self-protection inferences, more dissatisfaction and adverse effects, more abandonment, task avoidance, lack of cognitive involvement, and apathy. These externally-induced self-reactions complete the self-regulatory model. High satisfaction levels promoted by *proactive* (*externally-regulatory*) contexts involve learners in several kinds of self-motivation to keep on making an effort and to maintain their ideas of self-efficacy. In addition, they entail adaptive inferences associated with learning to plan and with scheduling targets necessary for the future. Low satisfaction levels associated with *reactive (a-regulatory)*, and *de-regulatory* contexts, however, erodes students’ motivation to persevere and their possibilities of adaptation, as well as the quality of their efforts to learn.

#### Principle 4. Self-Regulated Learning (SRL) as an Internally (SR) and Externally (ER) Mediated Process:

This principle proposes that *Self-Regulated Learning* (SRL) is a probabilistic process, mediated both internally (*Self-Regulation;* SR) and externally (*Externally-Regulation*; ER). For this reason, a person’s SRL may be explained and is predictable, as much because of the SR person’s characteristics as because of the characteristics of the context (ER), combined. This principle envisions human learning as a *combination* of the self-regulatory ability of the person (SR) and the externally-regulatory features of the context (ER), in which four types of interactions may occur. As may be observed, there is a difference between this model and the one described above, for it assumes that a person’s self-regulation is previous to and independent of self-regulation in learning. See **Table 3**.

**Table 3:** Types of combination among levels of variables in the Theory of Self- vs. Externally-Regulated Learning.

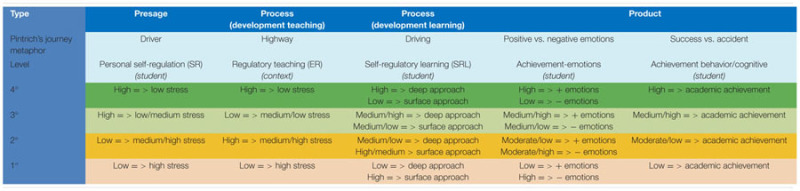

In order to better understand this concept, see Pintrich’s *journey metaphor* (Pintrich, 2000a,b; de la Fuente, 2004). A good or a bad driver (with the corresponding level of self-regulation, a-regulation, or de-regulation) together with a good or a bad road (with good, bad or no traffic signs) can vary the probability of having an uneventful drive or of having an accident. Therefore it is plausible to predict that (1) on a badly signed road (de-regulated) or on one with no signs (a-regulated), the level of expertise required (self-regulation) of the driver will be greater, whereas on a road with good signs (hetero-regulated or conducive to self-regulation), it is easier for the driver to maintain a high level of self-regulation; (2) however, neither of these two factors on their own guarantee a good journey. They must combine in the best possible way so that the journey takes place in optimum conditions.

Therefore, although it is true that the context may be actively chosen by the person, that is, redesigned or selected proactively, to favor self-regulation, it is also true that context has its own value in promoting to a greater or lesser extent self-regulatory behavior. Similarly, it may occur that one cannot bring about directing change in the kind of context. It seems reasonable to assume that (1) it is more difficult for a driver (even though s/he is an expert with a good level of self-regulation) to exercise self-regulation when driving on a road which has no traffic signs (a-regulatory) or which is designed to encourage speed (de-regulatory) than when s/he drives along a route with good road signs (hetero-regulatory), promoting self-regulation. In the field of *health*, (2) we may assume that a person who exercises self-regulatory behavior in alcohol consumption will find it more difficult to do so when s/he exposes him/herself to de-regulatory contexts (friends who all drink and who put social pressure on him/her) than when these contexts are regulatory and promote health (friends who do sport and who encourage not drinking socially).

### Application to the Psycho-educational Context

#### Self-regulation as a Meta-ability Student

*Self-regulation (SR)* Brown (1998, p. 62) saw self-regulation as an individual’s capacity to “plan, monitor and direct his or her behavior in changing situations.” Basically, this perspective involves periods of planning, of monitoring and of reflective assessment of one’s behavior. Self-regulation comes into play in several appraisal procedures amongst which are the management of feelings, reflections, and acts directed toward administering or restricting conduct (Blod, 2012). Self-regulation is a concept employed largely in the health arena (Baumeister et al., 1994; Baumeister and Vohs, 2007), but in the wake of Zimmerman’s (1989) assertion that such processes are to be found in many areas, researchers have begun to examine self-regulation elements present in many domains, such as education and work. Until then, self-regulation had been linked principally to addictive behaviors (Brown and Newby-Clark, 2005).

#### Externally Regulated Learning (ERL) As Effective Teaching

*Effective teaching* involves designing and developing a teaching context that encourages students to have the necessary stimuli and aids to respond with a good level of cognitive, emotional and behavioral commitment. This has several components of teaching process: motivation, atmosphere and the promotion of specific teaching and learning activities (Biggs, 2006). The *Regulated Teaching model* postulates diverse teaching strategies (de la Fuente and Justicia, 2007, p. 540): (a) evaluation with diagnostic and process-related; (b) giving information to the students about the teaching process and the structuring of learning activities; (c) setting the stage for self-regulation in the students. The teaching process is understood to be regulated when activities of teaching. Before beginning to explain what we understand by *regulatory teaching*, it is useful to determine the nature of *effective teaching*, the characteristics and dimensions which guide it, without overlooking the definition of an effective teacher, which is a very important concept when understanding both effective teaching and regulatory teaching (Lindblom-Ylänne et al., 2011; Roehrig et al., 2012).

This concept of effective teaching is closely related to *regulatory teaching* (RT). We cannot conceive of effective teaching without taking regulation into account. In both Biggs’s Model (Biggs, 2001) and the DEDEPRO Model (de la Fuente and Justicia, 2007; de la Fuente, 2011), regulatory teaching is a process variable, meaning that well-designed teaching and support help to pave the way for self-regulated learning on the part of the student (Kramarski and Michalsky, 2009). Here we mean that the teacher should be able to externally-regulate the learning process so that s/he may bolster the self-regulation of the learner him- or herself (Randi, 2004). de la Fuente and Justicia (2003) proposed that teaching is regulatory when teaching, learning and assessment are intextricably intertwined in the realization of independent, creative, harmonious and varied learning. This kind of regulation in teaching is twofold: it works well both for learning specific activities (*micro-regulation*) and for learning in a global activities (*macro-regulation)*. Thus, the teacher strives to instruct students to learn in a particular way, not in one direction but rather interactively, and pivoting on learners’ needs or abilities. The instructor should purposely teach bearing this in mind. The teacher’s mere presence in the teaching-learning process is not sufficient; s/he must shape it from the perspectives of concepts, timing, materials and procedures (de la Fuente and Justicia, 2007).

#### Combination between the Level of SR and Level of ER in Educational Contexts

*Self-Regulation* as a personal characteristic of the individual is a variable or a previous factor, present at the threshold of the learning situation (de la Fuente et al., 2014a, pp 604–605).

(1) *Type 1* combination (low-grade). The student has a low level of self-regulation (presage) and receives a low level of regulatory teaching. It will involve a low level of deep focus and positive emotionality, such as resilience, commitment and trust (process). He will experience a high level of negative emotionality, such as test anxiety and, ultimately, poor performance (product).
(2) *Type 2* combination (medium-low grade). The student has a low level of self-regulation (presage) and receives a high level of regulatory teaching. Therefore, the student will engage in a low/moderate level of deep focus with low/moderate levels of positive emotions, such as resilience, commitment, and trust. He will experience a moderate/high level of negative emotionality, such as test anxiety (process), ultimately performing at a moderate-low level (product).
(3) *Type 3* combination (medium-high grade). The student has a high personal self-regulation (presage) and receives a low regulatory teaching, will engage in a moderately high level of deep focus, with moderate/high levels of positive emotions, such as resistance, commitment and confidence, and low/moderate levels negative emotion, such as test anxiety (process), ultimately producing a moderately high level of performance (output).
(4) *Type 4* combination (high grade). The learner has high-grade personal self-regulation (presage) and receives high-grade regulatory teaching, he will engage in a highly deep approach with high levels of positive emotionality, such as resilience, engagement, and confidence, and a low level of negative emotionality, such as test anxiety (process), ultimately producing a high level of performance (product). See **Table 3**.

#### Level of Domain Theory: Micro-, Molecular, and Molar Analysis

This theory may be situated both in the domain of micro-analysis and at the level of molar and molecular analysis, called macro- or micro-regulation of the teaching -learning process. It assumes that the operationalization of the teaching and learning processes is equally important at the macro- and at the micro-levels of analysis. The regulation of the teaching process has two levels (de la Fuente and Justicia, 2007, pp 542–543):

##### Micro-regulation of learning (micro-analysis level)

**FIGURE 1:**
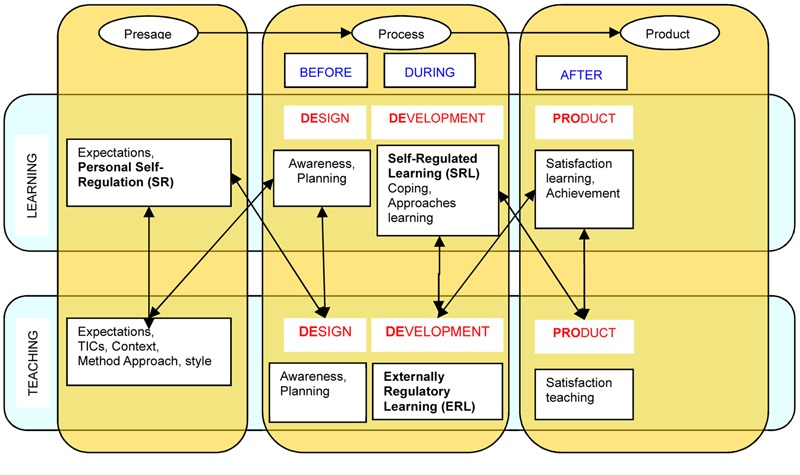
Variables of the Theory of Self- vs. Externally-Regulated Learning (SRL vs. ERL) in the context of the 3P and DEDEPRO model (de la Fuente and Justicia, 2007, p. 545).

This theoretical formulation may be considered in a domain at the level of molecular or microanalysis in the learning process (Cleary, 2011), as it establishes in an operative way the discrete behaviors of self-regulatory behavior during learning. **Figure 1** reflects its scope, specifically in the context of a more holistic or molar vision of the teaching-learning process.

##### Micro-regulation of teaching (micro-analysis level)

In which/by which we consider the instructional process variables that are carried forward by the individual teacher, who carries out certain teaching activities, for example, how to find the solution to a science problem or how to draft a written assignment.

##### Macro-regulation of teaching (at the level of molecular and molar analysis)

This is regarded as the regulation of the teaching process from a much wider perspective, considering the period of time that may be needed to bring it to fruition (a study plan, be it multi-year, annual, semester-long, trimester-long, monthly, or daily). Despite its importance, such macro-regulation has not been investigated sufficiently. However, studies on self-regulation are beginning to address this as an emerging topic, since regulatory teaching substantially encourages students’ self-regulation. See **Figure 1**.

## Evidence

### Role of Self-regulation

#### Self-regulation and Education

de la Fuente and Cardelle-Elawar (2011, p. 3) defined it as a student variable which “determines the level of effort that students will sustain in the process of active learning for the completion of a given task.” Results also indicated that varying degrees of personal self-regulation give rise to different kinds of coping strategies. When confronted with a perturbing scenario, learners who have a high degree of personal self-regulation display coping strategies which home in on the problem itself, while those whose level of personal self-regulation is low tend to deal with the situation in a more emotional way. A previous study (de la Fuente et al., 2008), in which students from compulsory secondary education participated, demonstrated that degrees of total personal self-regulation influenced how these learners understood the social ambience of their high school. More precisely, low and high degrees of total self-regulation were associated with students’ awareness of levels of good or bad conduct at school.

Recent research has pointed to associations among personal self-regulation, self-regulation in learning, coping strategies employed, and satisfaction in learning (McPherson et al., 2013; de la Fuente et al., 2015b; Zapata, 2013, Unpublished). Connections among self-regulation, learning approaches, coping strategies, and resilience in university students have also come to light (de la Fuente et al., 2015b, 2017a). In the case of academic emotions, the role of self-regulation in issues such as bullying and engagement/burnout has also been observed (de la Fuente et al., 2014b).

#### Self-regulation (SR) and Health

As we have already pointed out, a considerable part of healthcare research has taken the variable of personal self-regulation into account (Dempsey and Kauffman, 2017). Within this area, addictions especially have been investigated in association with this variable as they constitute such a serious social issue today. This research supports that self-regulation is a critical factor in substance abuse or abstinence (Muraven et al., 2002, 2005). These researchers discovered that lower levels of self-regulation was linked to higher alcohol intake and to more intense distress. Tangney et al. (2004) observed that more self-regulation level was associated with less alcohol abuse, with a higher grade point average, with better psychological and emotional adjustment, as well as with optimal responses. They found that changes in self-regulation and in self-efficacy were significantly predictive of the probability of abstinence (Chavarria et al., 2012). Furthermore, changes in self-regulation and in self-efficacy were largely independent of each other. This indicates that poorer levels of personal self-regulation contributed to the risk of suffering alcohol-related experiences, and of extending the duration of these effects (Hustad et al., 2009).

### Combined Effects of Self-regulation (SR) and Externally-regulated Learning (ERL) in the Self-regulated Learning (SRL)

Partial previous evidence supports the model, although more research should be carried out involving the new concepts proposed. Azevedo et al. (2007b) published a study in which they clearly illustrated this interaction in the field of technology teaching and learning, and found that the *SRL* condition gave rise to fewer learning effects than did the ERL condition. Later it was found that self-regulation is a presage factor which influences the perception of the teaching-learning process (de la Fuente et al., 2011).

Recent research has supplied empirical evidence for the typology of the interactions proposed in the model (de la Fuente et al., 2014a, 2015b,d,e, 2017b). Research has also showed how the self-regulation level and the stress level of the context together determine coping strategies in university students (de la Fuente et al., 2016b), and that the level of regulatory teaching, as an external regulatory variable, is associated with the level of academic emotions in learners (de la Fuente et al., 2016a). Finally, it has been demonstrated how training in effective or regulatory teaching brings about an improvement in the perception of the teaching process in university students (de la Fuente et al., 2013).

### Assessment in a Combined and Interactive Format

This theoretical formulation requires instruments which will allow for the combined assessment of the different levels of regulation outlined. Some already exist, while others are yet to be developed.

#### Self-regulation Assessment

Brown et al. (1999) designed the *Self-Regulation Questionnaire* (SRQ) to evaluate self-regulation. After, more research they went on to design a more concise scale, the *Short Self-Regulation Questionnaire* (SSRQ), which was validated in a study involving Spanish participants (Pichardo et al., 2013; Garzón et al., 2017). Results demonstrated the goodness of fit into four factors (goal planning, perseverance, decision making, and learning from mistakes.

#### Interactive Assessment of Regulatory Teaching and of Self-regulated Learning

The *Interactive Assessment of the Teaching and Learning Process* (IATLP) (de la Fuente and Martínez-Vicente, 2007) measures teaching and learning, and proposes feasible causal associations among presage-process-product variables. The IATLP is a self-report *scale/questionnaire* which collects both teacher and student responses, and is published in both Spanish and English. Variations in results observed suggest that this scale offers insights into the repercussions that the setting may have on the teaching and learning process (de la Fuente et al., 2012).

## Goodness and Limitations

Essential *goodnesses* of this theoretical formulation are:

(1) It adds the same phases of Externally Regulation (ER) to the original cyclical phases of the Self-Regulation (SR), and Self-Regulated Learning (SRL), allowing for a greater understanding of the teaching-learning binomial.
(2) It enables us to hypothesize in a probabilistic and parsimonious way the combined effects of *personal* characteristics (low-moderate-high levels of SR) and *contextual* characteristics (low-moderate-high levels of contextual ER). It introduces new concepts or regulation levels in the individual: *self-regulation, a-regulation*, and *de-regulación*, applicable to individuals and to contexts. It introduces new concepts regarding the external regulation levels of the context. In this way, a *externally-regulatory* context refers to the characteristics of the context and of the people within it, which promotes and makes self-regulation more probable. An *a-regulatory* context does not aid self-regulation. A *de-regulatory* context impedes self-regulation pro-actively.
(3) It is a model that is sufficiently holistic as to be applied to the analysis and study of diverse situations and human teaching-learning contexts, such as school, the family, road safety education, and health education.

The principal *limitations* of this theoretical model are:

(1) Its predictions must be demonstrated empirically, through more research in different contexts. Several recent research reports have shown empirically the relationships proposed, through models of linear association (de la Fuente et al., 2014a, 2016a,b), as well as non-linear ones (de la Fuente et al., 2017b), with promising results, in academic contexts.
(2) The theoretical model must be generalized to other areas, through the analysis of formal teaching-learning processes (schools), non-formal ones (family and associations), and informal ones (leisure time, internet use, and social networks), as well as to diverse areas in *education* (learning difficulties, prevention of bullying), in *health care* (prevention of alcohol abuse or of unwanted pregnancies; dealing with chronic diseases), and in *road safety* education (accident prevention).
(3) Also it is necessary to devise new assessment instruments and intervention programs, focused on contexts and on individuals, with reliable and valid methodologies for evaluating both kinds of constructs, at different ages and in different educational contexts, and especially for evaluating the degree to which different contexts are hetero-regulatory, a-regulatory, or de-regulatory as far as the active promotion of self-regulation in individuals is concerned, beyond their personal characteristics.

## Applicability

### General Applicability

The development of this theory is applicable in diverse contexts of combinatory behavior among internal and external human factors in which processes of learning and of teaching occur.

In *formal educational* processes, this theory enables us to know and analyze the weighting of personal characteristics (in self-regulation) and of contextual aspects (externally-regulation), when we attempt to explain self-regulatory behavior during learning at school or in other academic situations (Vermunt, 1989; Winne, 2001, 2005; Azevedo et al., 2007a; Winne and Hadwin, 2008; Hadwin et al., 2010; Vermunt and Donche, 2017). This evidence will allow us to analyze the causes of learning problems/difficulties, from interactive and complementary viewpoints, without bias exclusively toward students who learn successfully (Craig et al., 2011). To this end, we may employ e-assessment and e-intervention technological resources (de la Fuente et al., 2015a).

In *informal educational* processes or in those within the family, this theory may be used in the analysis of different problems, in the assessment and counseling of parents, as well as in the design of more regulatory contexts, promoting self-regulation in their children, so as to help them to progress from hetero-regulation to self-regulation (Bernier et al., 2010).

In *non-formal educational* processes (television, the internet, leisure activities...) this theory will be help to establish whether the context favors self-regulation, or if, on the contrary, it is a-regulatory or de-regulatory.

### Specific Applicability

In the field of *health care education*, it constrains the role of personal and contextual variables in assessment and interventions to improve self-regulatory behavior, taking into account the limitations encountered in the self-regulatory behaviors of individuals (Goudas et al., 2013; Mann et al., 2013). There are numerous reports on these kinds of interventions (Brownlee et al., 2000; Mann et al., 2013), but most are focused on the individual, in detriment to the context which may probabilize self-regulatory behavior. In addition, in the area of adherence to treatment, the role of self-regulation has been revealed consistently (Williams et al., 1998), without sufficiently taking into consideration external regulation factors. Another aspect worthy of note is the theory’s applicability to self-medication, in which external regulatory strategies are essential (Khantzian, 1997). No less important is its applicability in preventative education regarding alcohol consumption, in which self-regulatory mechanisms have been shown to be important (de la Fuente and Cubero, 2013; Marcos, 2013) without taking sufficiently into account external a-regulatory or de-regulatory contextual factors.

In the field of *road safety education*, this theory will help to assess de-regulatory behaviors and contexts, while at the same time allowing interventions both to improve self-regulatory behaviors (Gwyther and Holland, 2012), and to promote hetero-regulatory contexts which will probabilize self-regulatory behaviors while driving. In education for addiction prevention, this theory is especially useful in the light of the escalation in addictions to the internet (Kuss et al., 2014) and to alcohol, in which not only the role of self-regulation is important (Quinn and Fromme, 2010) but also the regulatory context. In *clinical* or *psychopathological* contexts, it may be a significant tool in self-regulation therapies (Kohen and Olness, 1996) to provide persons with self-regulatory meta-skills, and it will enable us to design hetero-regulatory contexts for the promotion of personal self-regulation. Of special note is relationship and marriage counseling, which has traditionally been approached from a self-regulatory perspective (Finkel et al., 2009) and which could be analyzed within a more interactive focus. In the area of *chronic illnesses*, it is crucial to consider the importance of self-regulation in patients and the probabilistic value of the *context* in guaranteeing adherence to the treatment, and, therefore, in the treatment of such health problems, beyond the self-regulation level of patients (Clark, 2013).

## Conclusion and Future Research

This new conceptual formulation enables us to approach in a more holistic and combined way with both internal and external variables, and interactions implicit in human learning processes in their interaction with contexts. It allows for re-balancing the role of the individual-context interaction, conceptually delving more deeply into the combinatory and interactive possibilities of both (self-regulatory, a-regulatory, and de-regulatory), without losing sight of the original, cyclical concept of self-regulation in Zimmerman’s model (2001). In a complementary way, this formulation allows for not only (a) a *micro-analytical* level in assessment and intervention (the analysis of the cognitive and motivational-affective processes of a person’s learning in a given apprenticeship, as well as in specific training, as promoted in the classic SRL model, Bembenutty, 2010, 2013; Herndona and Bembenutty, 2017), but also (b) evaluation and intervention at the *molecular level* (the analysis of the cognitive and motivational-affective processes which operate in a real given teaching-learning process, as established by the 3P and the DEDEPRO models, as well as at (c) the *molar level* (the analysis of teaching-learning processes which operate in non-formal and informal contexts, in face-to-face and virtual learning situations). What is more, this theoretical formulation not only enables us to analyze interactions which foster learning process in school and in other academic contexts – the preferential settings from the conceptual point of view of the *Theory of Learning* in classic studies of teaching-learning processes – but also it is a sufficiently ample and general conceptual framework to enable us to understand how contexts and individuals operate in different contexts of interaction. That is to say, it means that we have a *new conceptual heuristic* for the combinatory analysis of possible interactions and their behavioral consequences for persons in numerous contexts of human learning, and not only in school or in other academic situations.

The new *SRL vs. ERL Theory* has recently revealed evidence in support of the relevance of the combined and interactive effect of (1) the characteristics of personal self-regulation, and (2) the externally regulatory role of different contexts in explaining and predicting meta-cognitive, motivational, and affective variables, as well as achievement (de la Fuente et al., 2015c). The theory needs to be developed through the creation of new assessment instruments, which will be able to apply its claims to potentially important fields, not only to education, but also to health care and prevention of diseases (Anderson et al., 2006), to road safety, or to any behavior under analysis in which regulation as an important behavioral variable is implicit in its dimensions, whether internal (Brownlee et al., 2000; Artuch-Garde et al., 2017), or external (Azevedo et al., 2007b; Bernier et al., 2010), overcoming the simplistic idea of internal and external regulation – from a person-situation analysis (Kanfer et al., 2010)- and advancing toward more subtle meanings, in line with the validation of the self-regulation, a-regulation and de-regulation concepts.

## Epilogue

“Behold, there went out a sower to sow: and it came to pass, as he sowed [the seed], some fell by the way side, and the fowls of the air came and devoured it up. And some fell on stony ground, where it had not much earth; and immediately it sprang up, because it had no depth of earth. But when the sun was up, it was scorched; and because it had no root, it withered away. And some fell among thorns, and the thorns grew up, and choked it, and it yielded no fruit. And other fell on good ground, and did yield fruit that sprang up and increased; and brought forth, some thirty, and some sixty, and some an hundred!” (Gospel of Saint Mark, 4).

Historically, knowledge of Psychology has not been far removed from knowledge of popular phenomenology which, based on everyday observation and experimentation, has served to devise conceptual frameworks on which to explain and predict principles of life and human behavior. One example of this is parables, as experiential and heuristic teaching tools of educational and psychosocial phenomena. We may consider that in the parable of the sower, the seed refers to personal characteristics, while the earth where it falls refers to the role of the context of learning and development. And although Psychology does not operate with parables but with conceptual foundations, theoretical principles, predictions and empirical evidences, we consider that there is a certain conceptual similitude between predictions established in this parable and those deduced from the concept of the theory itself which we shall go on to present. For this reason, we may define *Theory of Self- vs. Externally-Regulated Learning*^TM^ (de la Fuente et al., 2015b) as a scientific or heuristic version explaining the *parable of the sower*, cited above.

## Dedication

This article is dedicated to Professor Emeritus B. J. Zimmerman (City University of New York), with thanks for his conceptual formulations which have changed our way of understanding *human learning*, in recognition of his SRL Theory.

## Author Contributions

The author confirms being the sole contributor of this work and approved it for publication.

## Conflict of Interest Statement

The author declares that the research was conducted in the absence of any commercial or financial relationships that could be construed as a potential conflict of interest.
